# Missing Data Imputation of Solar Radiation Data under Different Atmospheric Conditions

**DOI:** 10.3390/s141120382

**Published:** 2014-10-29

**Authors:** Concepción Crespo Turrado, María del Carmen Meizoso López, Fernando Sánchez Lasheras, Benigno Antonio Rodríguez Gómez, José Luis Calvo Rollé, Francisco Javier de Cos Juez

**Affiliations:** 1 Maintenance Department, University of Oviedo, San Francisco 3, Oviedo 3307, Spain; E-Mail: ccrespo@uniovi.es; 2 Departamento de Ingeniería Industrial, University of A Coruña, A Coruña 15405, Spain; E-Mails: mmeizoso@udc.es (M.C.M.L.); benigno@udc.es (B.A.R.G.); jlcalvo@udc.es (J.L.C.R.); 3 Department of Construction and Manufacturing Engineering, University of Oviedo, Gijón 33204, Spain; 4 Project Management Area, Mining Department, University of Oviedo, Oviedo 33004, Spain; E-Mail: fjcos@uniovi.es

**Keywords:** missing data imputation, multivariate imputation by chained equations (MICE), multiple linear regression, solar radiation, pyranometer

## Abstract

Global solar broadband irradiance on a planar surface is measured at weather stations by pyranometers. In the case of the present research, solar radiation values from nine meteorological stations of the MeteoGalicia real-time observational network, captured and stored every ten minutes, are considered. In this kind of record, the lack of data and/or the presence of wrong values adversely affects any time series study. Consequently, when this occurs, a data imputation process must be performed in order to replace missing data with estimated values. This paper aims to evaluate the multivariate imputation of ten-minute scale data by means of the chained equations method (MICE). This method allows the network itself to impute the missing or wrong data of a solar radiation sensor, by using either all or just a group of the measurements of the remaining sensors. Very good results have been obtained with the MICE method in comparison with other methods employed in this field such as Inverse Distance Weighting (IDW) and Multiple Linear Regression (MLR). The average RMSE value of the predictions for the MICE algorithm was 13.37% while that for the MLR it was 28.19%, and 31.68% for the IDW.

## 1. Introduction

A meteorological or climate observation network is composed of a set of weather stations. They are usually placed at isolated points of a geographical zone, in order to determine the values of the meteorology and climatology variables of that area. Multiple variables such as air temperature, atmospheric pressure, wind speed and direction, relative humidity, rainfall, solar radiation, *etc.*, are measured and registered by each station and finally the data is sent to a central database of the network to be processed and stored [1].

Failures in the measurement process may occur for any variable, and consequently lack of and/or incorrect data can appear. These errors can often be detected, although only sometimes corrected, since the more variable the measurements are, the more erroneous or less precise the data imputation results.

Solar irradiation records in particular depend on the combined effects of both astronomical and meteorological events. Atmospheric conditions modify the extraterrestrial solar irradiation in such an ostensibly random manner that the global solar irradiation on the horizontal surface presents evolution randomness, with temporal and spatial variations due to weather conditions [2].

The amount of solar energy incident on the ground depends heavily on the state of the sky. Previous research has reported that among all the variables causing heterogeneity of solar radiation in the ground, atmospheric conditions such as cloud cover [3], aerosols and water vapor are the most important. In the case of cloud cover, factors such as cloud base height, cloud evaporation and formation and velocity have been reported as important [4]. In the case of aerosols a considerable reduction in the UV intensity has been observed during periods of high aerosol loading [5]. Previous research also demonstrated that the more water vapor solar radiation finds, the smaller the amount of solar energy present on the ground [6].

Global radiation includes radiation received directly from the solid angle of the sun's disc, as well as diffuse sky radiation that has been scattered in traversing the atmosphere. Global solar radiation (G) measurements at ground level are made primarily with pyranometers that use thermo-electric, photoelectric, pyro-electric or bimetallic elements as sensors [7].

The World Meteorological Organization (WMO) classifies pyranometers as secondary standard, first class and second class meters according to their measurement performance characteristics, such as spectral range, sensitivity, directional response, non-stability, temperature response, response time and non-linearity among others. Meteorological networks do not have the same type of equipment in all of their weather stations and, consequently, the radiation measurement quality varies from one to another. The diversity of the instruments installed with different accuracy levels, together with the need for the frequent instrument calibration, makes it very difficult to achieve a homogeneous data base [8].

The following problems have been reported [9] for solar global radiation: No signal from the sensor, an unstable signal, a lower or higher signal than physical limits, data not collected or stored and diurnal profiles systematically asymmetric with respect to the solar noon, among others. The following can be noted as likely reasons for such failures [10,11]: A damaged cable or with corrosion; the loss of proper electrical grounding; alterations in programs of data logger systems; moisture inside an element of the pyranometer; reflected radiation from properly-positioned towering cumulus clouds exceeding the solar constant for periods of less than ten minutes; half-melted frost or snow on the dome; communications failure, *etc.*

Some of these causes are temporary and may disappear spontaneously, but others require the intervention of a maintenance task force, and therefore errors persist for different periods of time. Lack of data or the presence of erroneous data adversely affects the study of any time series. For instance, solar energy applications need continuous radiation data time series to correctly assess the usefulness of the particular application and in its implementation, so additional procedures have been established to fill in missing values (where data is initially lacking or has been removed via quality checks) in the time series of solar radiation data [12]. Consequently, a process of data imputation may be followed for filling data series with estimated values.

Different criteria can be applied in order to obtain a missing value or a set of missing values in a series of solar irradiation data (G). Deterministic, random or mixed methods are available for this purpose. Some specific examples are discussed below.

Physical models such as MRM [13], ESRA [14], REST2 [15] or SIRAMix [16], which account for the estimated solar irradiation in terms of physical variables (aerosols, precipitable water, turbidity coefficients, total ozone in the vertical column, dry-bulb temperature, site pressure, *etc.*) are a good solution when the values of these auxiliary variables are known. The main advantage offered by these models is their spatial independence. In addition they do not require solar radiation data measured at the Earth's surface. However, the physical methods need complementary meteorological data to characterize the interactions of solar radiation with the atmosphere. SAs an example we can cite the ESRA method, which needs five inputs or the REST2 method, which needs a total of 10 inputs. Other methods that estimate solar radiation form satellite images have been used and tested by several authors [17,18].

Correlation relationships can be established as a second approximation. If weather stations are sufficiently close together, the difference in G records should be small, and then the space distance criteria can be applied. This argument is valid for clear or cloudy skies but performs worse for partly cloudy skies over short time scales. Also, when the missing data is only one, among known data, a simple interpolation can be the best solution [19].

Autoregressive properties of the signal allow us to take into account more separated values to auto-complete the series. ARIMA techniques were used to forecast solar radiation time series [20], so ARIMA models can be used to impute data in time series.

A similar study in terms of number of stations and time scales has been carried out [21], although in a different geographic area. This study proved that it was better to interpolate sequences of up to four missing values, and the first and the last missing values in longer sequences, using data from the same site. Otherwise it is better to use simultaneous data from the other sites of the network.

All the methods' performance is influenced by the time granularity. Weather stations provide data in several time scales: measuring equipment supplying data in the lowest scale and upper scales are built based on it. Normally, missing values in longer scales are estimated with less error than in shorter ones.

Several studies used Inverse Distance Weighting (IDW) to estimate solar irradiation [22–24], and also to interpolate other variables like temperature or precipitation [25–27].

Techniques for estimating data are essentially applications of statistics, but they should also rely on the physical properties of the system under consideration. IDW is a function based on one parameter, *i.e.*, distance, and it assumes that the region has a uniform characteristic [26]. A “cut-off” criterion is often used to limit either the distance to the locations considered or the number of observations considered [28].

In [22], working with hourly observations, it was concluded that the interpolations for distances beyond 34 km show an RMSE over 25% and it is suggested that in this case satellite measurements are more accurate. Consequently, the selected geographical area for this study is a small zone with a high density of measurement locations. The maximum distance between stations is less than 20 km.

In [23], working with monthly mean values, the distances between stations are over 95 km, and the relative error is under 29%. Finally in [24], the authors worked with 15-min observations but the RMSE was obtained by calculating daily aggregations and eliminating days identified as atypical. The values for RMSE are between 26% and 40%. Random errors tend to decrease when the data are averaged over a particular time period.

Kriging is a spatial interpolation approach that has been applied to estimate monthly irradiation [29]. However, the low number of stations and the high variability among the ten-minute scale data advise against the use of this method.

Regression models have been widely used for the estimation of global solar radiation. The most frequently selected variable is sunshine duration, where the Angstrom-Prescott-Page model is the main exponent for the monthly average daily global radiation [30]. Air temperature also appears in many models, being the Bristow-Campbell being one of the most widely-used models due to its simplicity and the availability of input data [31,32]. However, in most cases, the estimated radiation is a daily or monthly average daily value, and to our knowledge there are no sub-hourly regression models using temperature.

Cloud cover, relative humidity or wind velocity are other variables used to estimate solar radiation [33,34]. In [33], a method is developed to estimate hourly solar radiation by using relative humidity and ambient temperature in order to obtain a matrix of atmospheric transmittance coefficients that need to be adjusted to the particular area. The RMSE of this estimation method is 8.3%. The reason for this low value is that the method estimated hourly solar radiation. Time granularity is a key factor to compare the performance of the different methods. The longer the time scale, the fewer the errors. This reference is an example of a correlational method, but the error cannot be compared with the present research, as the authors used hourly data.

The aim of this paper is to evaluate a method which allows the network itself to fill the missing data of a sensor, using either all or just a group of the measurements of the remainder sensors. It is therefore necessary to work with ten-minute scale data, which makes it a challenge.

The rest of the paper is organized as follows: Section 2 includes a geographical description of the chosen study area and the dataset, and also gives the main characteristics of the sensors. Section 3, describes the three interpolation/imputation methods used and the validation criteria applied. Section 4 presents a comparison of the results achieved with each method. Finally the conclusions are drawn.

## 2. Experimental Section

### 2.1. Description of Study Area and Data

The current study uses ten-minute data collected from the MeteoGalicia network [29], a regional meteorological service with more than 100 locations located in Galicia (Spain) providing global radiation data. The network integrates stations with both meteorological and agro-climatic purposes and the regional government openly offers observations from its stations on the Internet [35].

**Figure 1.**
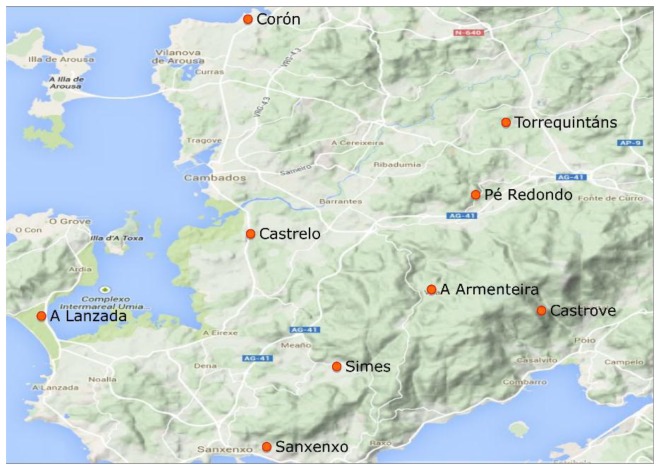
Map of the area studied.

The study area extends over a small area located in northwest Spain (Figure 1), between 42°24′–42°34′ northern parallels and 8°42′–8°52′ western meridians, covering an area of approximately 254 km^2^. Bordered by the Atlantic Ocean to the West, and situated between two coastal bays, called “rias” (Ría de Villagarcía and Ría de Pontevedra), it has a temperate maritime climate and is one of the Galician areas that receives more solar radiation [36].

Grapevine cultivation has a widespread presence in the region. This is the main reason for the high density of meteorological stations in the area. The dataset collected for this study comes from nine closely spaced meteorological stations. The greatest distance between them is less than 20 km. Table 1 shows the distance and the correlation coefficients matrix of the variable solar radiation for all the meteorological stations involved in the present study.

Geographical distribution and some climatological parameters (yearly mean values for 2013) of the studied radiometric stations are presented in Table 2. The data collected represents the global horizontal solar radiation (W·m^−2^) and is available on a 10-min scale basis, therefore, each station supplies 144 values per day.

The nine series ran from 21 December 2012 to 20 January 2014. The period of measurements was chosen to take into account seasonal variability [37]. Each station registered a maximum of 56,880 observations during this period, and the dataset includes a total of 511,345 observations. During the period of study, data missing for each station were: A Armenteira (1.00%), A Lanzada (0.004%), Sanxenxo (0.004%) and Corón (0.002%). No data was missing in the other stations.

### 2.2. Sensors for the Measurement of the Solar Radiation Flux Density: Pyranometers

**Figure 2.**
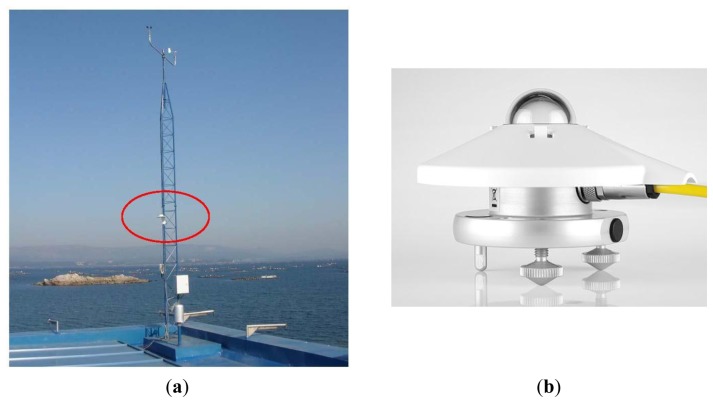
(**a**) Corón meteorological station with its thermopile pyranometer inside a circle. The model is the CMP-3 (source: www.meteogalicia.es); (**b**) Picture of the CMP-3 sensor taken from Kipp-Zonnen pyranometers brochure.

The nine stations employed in the present research have three different pyranometers. Table 3 shows the main properties which are of concern when evaluating the quality of these instruments. Figure 2 shows one pyranometer model and how it is placed in the meteorological station.

The thermopile detectors are sensitive to the whole shortwave spectrum in contrast to the solid-state silicon photodiodes. The basic uncertainties in the best practical solar radiation data available are roughly 5% in total global horizontal [38].

## 3. Methodology

In order to facilitate modelling, only daytime (sunrise to sunset) solar irradiance readings were considered. No other filter was applied to remove outliers. The MeteoGalicia network provides a flag to identify the quality of each value measured (see Table 4). In the dataset there is no data with codes: 0, 4, or 5, and some with codes 2, 3 and 9, but only original validated data (code 1) has been considered. Therefore the dataset was divided into a training set, with two thirds of the samples (14,553 samples); and a test set, with the remaining one third of the samples (7276 samples) as usual. The training set is used to generate the models by different methods (MLR, MICE); the test set is used to validate the models with independent data, since the test set did not provide information to be able to build the models.

### 3.1. Inverse Distance Weighting (IDW)

IDW is a deterministic method of spatial interpolation. It is based on the distance between the location for which a value has to be interpolated and the locations of observations [28]. The point is that the solar radiation in a particular location presents a high correlation with the values registered in closed sites. Thus, it is possible to estimate solar radiation at any point through a linear combination of the values measured from neighboring sites. If G_E_ is the solar irradiance estimation at a site with no measurement, it can be calculated following the Equations (1) and (2) [23]:

(1) $$G_{E}=\frac{\sum_{i=1}^{n}W\left(r_{i,E}\right)G_{i}}{\sum_{i=1}^{n}W\left(r_{i,E}\right)}$$

(2) $$W\left(r_{i,E}\right)=\frac{1}{r_{i,E}^{p}}$$

where *G_i_* is the record of solar measured irradiation at site “*i*”, with *i* = 1, 2, …, *n*, *W*(*r_i,E_*) is the weighting function between the *i*-th site, *r_i,E_* is the distance between the *i*-th solar irradiation measurement station and the estimation site, and “p” is the power parameter used in the interpolation. *p* = 2 is often chosen to provide even more weight to the closest locations [28]. We considered three cases: *p* = 1, 1.5, 2. Altitude was not taken into account for the distance calculations because the stations are all at practically the same height above sea level.

### 3.2. Multiple Linear Regressions Models (MLR Models)

The MLR models use a set of independent variables that helps to explain the independent variable; in this case, the measures of the neighboring stations were chosen as explanatory variables because of the high correlations between them (see Table 1). The correlation coefficient (CC) is expressed according to the following equation:

(3) $$CC=\frac{\sum_{i=1}^{n}(Gx_{i}-\bar{Gx_{i}})(Gy_{i}-\bar{Gy_{i}})}{\sqrt{\sum_{i=1}^{n}{\left(Gx_{i}-\bar{Gx_{i}}\right)}^{2}}\sum_{i=1}^{n}{\left(Gy_{i}-\bar{Gy_{i}}\right)}^{2}}$$

where *Gx_i_* and *Gy_i_* are the ten-minute measurements of global irradiation at the stations *x* and *y* respectively, and ${\bar{Gx}}_{i}$ or ${\bar{Gy}}_{i}$ are the mean of all the measures.

Multiple linear regression is a statistical method that accordingly models the relationship between a dependent variable (*y*) and a set of independent variables (*x*_1_, *x*_2_, …, *x_p_*). The model can be represented as follows [39]:

(4) $$y=\alpha +\beta_{1}x_{1}+\beta_{2}x_{2}+\ldots +\beta_{p}x_{p}+ɛ$$

where α is called the intercept, β*_i_* are called the slopes or coefficients, ε is an error with zero mean and constant variance, and it is accepted that each independent variable has a linear relationship with the dependent variable.

Equation (4) can be rewritten in matrix form, *i.e.*:

(5) $$\left(\begin{array}{c} y_{1}\\ y_{2}\\ \vdots \\ y_{n} \end{array}\right)=\left(\begin{array}{ccccc} 1 & x_{11} & x_{12} & \cdots & x_{1p}\\ \vdots & & \ddots & & \vdots \\ 1 & x_{n1} & x_{n2} & \cdots & x_{np} \end{array}\right)\;\left(\begin{array}{c} \alpha \\ \beta_{1}\\ \vdots \\ \beta_{p} \end{array}\right)+\left(\begin{array}{c} \in_{1}\\ \in_{2}\\ \vdots \\ \in_{n} \end{array}\right)$$

In this study *y_i_* are the ten-minute observations of one station, and *x_ij_* are the ten-minute observations of the remaining eight. Therefore in this case *n* = *p* = 9 and β*_p_* are the coefficient associated to location “*p*”. In order to obtain the intercept and the coefficients we took the least square approach with a confidence interval of 95%. After attending to the values of *F* and *t* statistics, coefficients not significantly different from zero are set to zero for the model.

### 3.3. The MICE Algorithm

The Multiple Imputation by Chained Equations (MICE) algorithm developed by van Buuren and Groothuis-Oudshoorn [40] is a Markov Chain Monte Carlo Method where the state space is the collection of all imputed values. Like any other Markov Chain, in order to converge, the MICE algorithm needs to satisfy the three following properties [41–44]:

- *Irreducible*: The chain must be able to reach all parts of the state space;
- *Aperiodic*: The chain should not oscillate between different states;
- *Recurrence*: Any Markov chain can be considered as recurrent if the probability that the Markov chain starting from *i* will return to *i* is equal to one.

In practice, the convergence of the MICE algorithm is achieved after a relatively low number of iterations, usually somewhere between five and 20 [44]. According to the experience of the algorithm creator, in general five iterations are enough, but some special circumstances would require a greater number of iterations. In the case of the present research, and due to the performance of the results obtained when compared with the other methods applied, five iterations were considered to be enough. This number of iterations is much lower than in other applications of the Markov Chain Monte Carlo methods, which often require thousands of operations. In spite of these, and from a researcher's point of view and experience, it must be also remarked that in the most common of the applications each iteration of the MICE algorithm would take several minutes or even a few hours. Furthermore, the duration of each iteration is mainly linked with the number of variables involved in the calculus and not with the number of cases. It must be taken into consideration that imputed data can have a considerable amount of random noise, depending on the strength of the relations between the variables. So in those cases in which there are low correlations among variables or they are completely independent, the algorithm convergence will be faster. Finally, high rates of missing data (20% or more) would slow down the convergence process work. The MICE algorithm [44] for the imputation of multivariate missing data consist on the following steps:

1. Specify an imputation model $P(Y_{j}^{\text{mis}}|Y_{j}^{\text{obs}},Y_{-j},R)$ for variable *Y_j_* with *j*=1,…,*p*.
  The MICE algoritm obtains the posterior distribution of *R* by sampling interative from the above represented conditional formula. The parameters *R* are specific to the respective conditional densities and are not necessarily the product of a factorization of the true joint distribution.
2. For each *j*, fill in starting imputations $Y_{j}^{0}$ by random draws from $Y_{j}^{\text{obs}}$.
3. Repeat for *t*=1,…,*T* (iterations).
4. Repeat for *j*=1,…,*p* (variables).
5. Define $Y_{-j}^{t}=(Y_{1}^{t},\ldots ,Y_{j-1}^{t},Y_{j+1}^{t-1},\ldots ,Y_{p}^{t-1})$ as the currently complete data except *Y_j_*.
6. Draw $\emptyset_{j}^{t}\sim P\left(\emptyset_{j}^{t}|Y_{j}^{\text{obs}},Y_{-j}^{t},R\right)$.
7. Draw imputations $Y_{j}^{t}\sim P\left(Y_{j}^{\text{mis}}|Y_{j}^{\text{obs}},Y_{-j}^{t},R,\emptyset_{j}^{t}\right)$.
8. End repeat *j*.
9. End repeat *t*.

In the algoritm referred to, *Y* represents a *n* × *p* matrix of partially-observed sample data, *R* is a *n* × *p* matrix, 0–1 response indicators of *Y*, and ∅Ø represents the parameters space. Please note that in MICE imputation [45], initial guesses for all missing elements are provided for the *n* × *p* matrix of partially observed sample. For each variable with missing elements, the data are divided into two subsets, one of them containing all the missing data. The subset with all available data is regressed on all other variables. Then, the missing subset is predicted from the regression and the missing values are replaced with those obtained from the regression. This procedure is repeated for all variables with missing elements. After this, all the missing elements are imputed according to the algorithm explained above, the regression and predictions are repeated until the stop criterion is reached. In this case, until a certain number of consecutive iterates fall within the specified tolerance for each of the imputed values.

### 3.4. Validation of the Models

Leave-one-out cross-validation has been used to analyze the spatial error of interpolated data [24,25]. This procedure involves using eight of the nine stations in the model to obtain the estimated value in the ninth station (this one is left out) in order to calculate RMSE and MAE for this station. The process is repeated nine times, once for each station.

The performance of the three methods has been evaluated using common statistics: Root Mean Square Error (RMSE), Mean Absolute Error (MAE) both expressed in W·m^−2^, and in percentage of the measured mean values, *i.e.*:

(6) $$\text{RMSE}(Wm^{-2})=\overset{n}{\underset{i=1}{\text{∑}}}\sqrt{\frac{1}{n}{\left(\hat{G_{i}}-G_{i}\right)}^{2}}$$

(7) $$\text{MAE}(Wm^{-2})=\frac{1}{n}\overset{n}{\underset{i=1}{\text{∑}}}\left|\hat{G_{i}}-G_{i}\right|$$

(8) $$\text{RMSE}\left(\%\right)=\frac{\text{RMSE}}{\frac{1}{n}\sum_{i=1}^{n}G_{i}}x100$$

(9) $$\text{RMSE}\left(\%\right)=\frac{\text{RMSE}}{\frac{1}{n}\sum_{i=1}^{n}G_{i}}x100$$

where *G_i_* and *G_l_^* are the measurements and the model-estimated values of global radiation respectively, and “*n*” is the number of ten-minute data points of the validation set. The RMSE weights large estimation errors more strongly than small errors and it is considered a very important model validation metric. Also, MAE is a useful complement of the measured-modeled scatter plot near the 1-to-1 line [45].

## 4. Results and Discussion

In this section the results of the different models tested are presented in order to compare their performance.

### 4.1. Results of IDW Method

Table 5 shows the RMSE and MAE for the IDW model. In spite of the short distance between the radiometric stations, the IDW model offers the poorest results. The influence of the power parameter “*p*” is barely noticeable, even though it is true that the small value of RMSE is obtained in most cases for *p* = 2. Exceptions are Castrelo, Castrove and Sanxenxo, where the lowest RMSE is obtained for *p* = 1; and Corón and Torrequintáns, where this value is obtained for *p* = 1.5. The average difference between the maximum and minimum values obtained for each station with different “*p*” was less than 1%.

### 4.2. Results of MLR Models

In Table 6 the multiple linear regression models for each location are presented. As expected, for each model the highest coefficient is related with the station that had shown the highest correlation (see CC in Table 1), and in some cases the stations with lower correlations have disappeared from the model. However, it is clear that the nearest locations do not always have the highest weight within the total of locations; in fact, this only occurs in four of the stations: Pé Redondo, Castrove, Sanxenxo and Torrequintáns. This explains the similar RMSE obtained for these MLR models (see Table 7) and the corresponding IDW models. An exception was Sanxenxo, whose MLR model offers a significant improvement, as with A Armenteira's. A detailed review of MLR models for Sanxenxo and A Armenteira shows that each of them has a relatively high negative coefficient, which tends to compensate for the high values given to the other stations. In the preliminary performance tests carried out with the dataset of one of the locations of this study, the air temperature was added to the regression model. However, the result, in terms of RMSE, was only slightly better (2%) with the addition of this variable, so finally no auxiliary variables were added to the MLR models.

### 4.3. Results of MICE Method

Table 8 shows the RMSE and MAE values obtained by means of the MICE algorithm for the nine meteorological stations in the study. Due to the random component of this algorithm, the procedure was applied five times for each of the stations. In order to verify that the results obtained for the five different iterations are better than those achieved by the other methods, the five iterations are presented. These tables also contain the average values of the five replications.

### 4.4. Comparison of the Three Methods

Finally, Tables 9 and 10 show the RMSE and MAE values of the average MICE algorithm in comparison with the MLR and IDW methods. As may be observed in Table 9, the RMSE results of the MICE algorithm are much lower than those for the other two methods: the average RMSE value of the nine meteorological stations for the MICE algorithm was 13.37% while that for the MLR it was 28.19% and 31.68% for the IDW. It must be highlighted that in all the stations the RMSE values using the MICE algorithm were lower than in the MLR and IDW methods. Similarly, Table 10 shows how MAE values of the results of MICE algorithm are lower in all the stations than in MLR and IDW methods. The average value of the MAE with the MICE algorithm for the nine stations was 14.14%, while the average values for the same stations obtained with MLR and IDW methods were of 35.30% and 34.19% respectively.

All the calculi corresponding to the different algorithms were performed with a computer equipped with an Intel Xeon E5-1650 processor and 16 GB RAM. The average time of all the runs of the MICE algorithm was 334.66 s, with a standard deviation of 9.98 s. There were a few variations in the computer times, depending on the meteorological station estimated. The average time of the five MICE replications for each meteorological station was as follows: Castrelo 335.60 s, Simes 339.00 s, Armenteira 330.4 s, Pe Redondo 322.8 s, A Lanzada 328.6 s, Castrove 332.6 s, Sanxenxo 334.8 s, Corón 343.4 s and Torrequintans 334.7 s. The computational time required for the calculus of the results of IDW and MLR methods was always under one second in both cases.

## 5. Conclusions/Outlook

Solar radiation presents a very high variability at ten-minute scales and ostensibly random behavior in our geographical study area; hence data imputation is difficult when a datapoint or a set of data is lost. IDW and MLR methods show similar performance taking MAE and RMSE criteria. The use of auxiliary variables, such as temperature, does not represent a significant enhancement. The MICE algorithm performed better. In this paper the validity of the MICE method in imputing global solar radiation gaps has been demonstrated. In spite of its larger computational cost, better results were obtained by the MICE algorithm. It can therefore be stated that this algorithm is of great interest for all those applications not requiring an answer in real time.

The estimation of missing data is required for some statistical techniques such as time series analysis. These techniques enable prediction models to be created and improved. The interest in these kinds of models is multiple as they can be employed for the evaluation of the solar energy available in order to take technical decisions about the best solution for its exploitation, for the estimation of derivate variables such as evotranspiration and in combination with other parameters for the evaluation of harvest productivity.

Once they know the performance of the MICE for recovering lost data from nearby stations, the authors of the present research propose to evaluate the use of this method as an estimator of the ten-minute radiation values at those points placed in intermediate positions between stations where a solar radiation device is not permanently located.

In this case, the application of the MICE algorithm cannot be performed directly. The terrain must be taken into account to determine that horizon of the sun is visible where the estimate is made. By taking this into account, the MICE algorithm will impute only those values corresponding to a horizon clear of obstacles to the sun's trajectory.

This would make it possible to obtain historical series of solar radiation for any point where solar radiation is not directly measured. These historical series would be used by means of simulations in order to improve the calculus of the performance of photovoltaic facilities at any point it would be required. Finally, they could also be used to simulate the growth of crops, or in any other application in which the knowledge of the series of solar radiation is crucial.

## Figures and Tables

**Table 1.** Distances among the studied stations (km) and correlation coefficients (CC).

**Distances (Km)**								
Torrequintáns	8.84	12.18	7.95	3.43	15.84	8.70	16.36	8.70
0.91	Castrelo	6.56	5.71	6.60	7.01	8.93	9.80	9.87
0.90	0.89	Simes	4.44	8.81	8.66	6.32	4.19	16.16
0.89	0.87	0.95	A Armenteira	4.52	11.08	3.25	8.60	13.46
0.93	0.90	0.89	0.89	Pé Redondo	13.46	5.64	13.0	10.32
0.89	0.92	0.89	0.86	0.88	A Lanzada	14.12	8.76	14.83
0.90	0.88	0.90	0.90	0.89	0.88	Castrove	9.97	15.75
0.90	0.91	0.92	0.87	0.89	0.93	0.90	Sanxenxo	19.66
0.90	0.91	0.87	0.85	0.88	0.90	0.87	0.89	Corón

**Correlation Coefficients**								

**Table 2.** Geographical and climatological parameters for the stations studied.

**Station**	**Lat.**	**Long.**	**Elev**	**Temperature (°C)**			**Humidity**	**Precipitation**	**Irradiation G**	**Sunshine Hours**

	**(°)**	**(°)**	**(m)**	**Min.**	**Max**	**Mean**	**(%)**	**(L/m^2^)**	**(10 kJ/m^2^day)**	**(h)**
Castrelo	42.49	−8.80	32	5.0	24.8	14.6	84.1	128.8	1346.1	184.5
Simes	42.44	−8.77	97	4.8	26.0	14.7	80.4	147.7	1510.2	221.2
A Armenteira	42.47	−8.74	256	3.4	24.5	13.1	82.3	211.9	1135.7	152.8
Pé Redondo	42.51	−8.73	150	6.0	25.5	14.6	80.4	175.4	1246.1	166.1
A Lanzada	42.46	−8.88	9	5.7	25.0	14.7	80.1	128.1	1425.4	201.1
Castrove	42.46	−8.70	424	5.5	23.5	12.9	80.6	206.3	1338.2	180.8
Sanxenxo	42.41	−8.80	34	6.6	25.5	15	77.3	147.5	1464.8	188.3
Corón	42.58	−8.80	3	7.4	23.8	14.9	78.5	149.7	1453.5	199.3

**Torrequint**á**ns**	**42.54**	−**8.72**	**52**	**4.5**	**25.7**	**14.5**	**73.9**	**121.0**	**1318.8**	**175.5**

**Table 3.** Characteristics of the network pyranometers.

	**SKS 1110 Skye**	**SP-LITE 2 Kipp & Zonnen**	**CMP-3 Kipp & Zonnen**

**Sensor**	**Silicon Photocell**	**Photodiode**	**Thermopile**
Maximum operational irradiance	5000 W/m^2^	2000 W/m^2^	2000 W/m^2^
Spectral range	0.4–1.1 μm	0.4 to 1.1 μm	0.3–2.8 μm
Sensitivity	1 mV/100 W/m^2^	60–100 μV/W/m^2^	5 to 20 μV/W/m^2^
Directional response	5% max	<10 W/m^2^	<20 W/m^2^
Non-stability (Change/year)	±2%	<2%	<1%
Temperature response	±0.2%/°C	<0.15%/°C	<5% (−10 °C to +40 °C)
Response time	10 ns	<500 ns	<18 s
Non-linearity	<0.2%	<2.5% (0 to 1000 W/m^2^)	<1.5% (100 to 1000 W/m^2^)
Number of stations	5	3	1

**Table 4.** Quality flags of the measurements provided by the network.

**Quality Code**	**Description**
0	No validated data
1	Original validated data
2	Suspect data
3	Erroneous data
4	Accumulated data
5	Interpolated data
**9**	**No registered data**

**Table 5.** RMSE and MAE obtained with the IDW method.

	**RMSE (%)**			**MAE (%)**		
	
	***p* = 2**	***p* = 1.5**	***p* = 1**	***p* = 2**	***p* = 1.5**	***p* = 1**
Castrelo	29.90	29.49	29.13	23.03	22.93	22.89
Simes	31.55	32.09	32.87	35.81	37.54	39.66
A Armenteira	37.33	37.79	38.77	46.51	49.18	52.53
Pé Redondo	29.67	29.98	30.56	37.53	38.44	39.77
A Lanzada	30.41	30.61	30.84	24.31	24.58	24.88
Castrove	35.84	34.87	34.34	44.05	46.90	50.29
Sanxenxo	34.88	33.93	33.25	31.51	31.89	32.54
Corón	32.70	32.69	32.73	29.13	29.12	29.17
Torrequintáns	27.21	26.73	26.87	28.50	28.69	29.32

**Table 6.** Multiple Linear Regression Models for each location.

**Model Parameters**	**Castrelo**	**Simes**	**A Armenteira**	**Pé Redondo**	**A Lanzada**	**Castrove**	**Sanxenxo**	**Corón**	**Torrequintáns**
Interception	8.57	0	−8.41	7.45	13.30	−3.26	−2.50	23.23	2.10
β_1_ (Castrelo)	-	0.06	0	0.16	0.31	0	0.16	0.27	0.11
β_2_ (Simes)	0.09	-	0.69	−0.07	0	0.03	0.47	0	0.06
β_3_ (A Armenteira)	0	0.60	-	0.21	0	0.37	−0.21	0	0.08
β_4_ (Pé Redondo)	0.17	−0.05	0.18	-	0.04	0.14	0.03	0.09	0.41
β_5_ (A Lanzada)	0.27	0	0	0.03	-	0.08	0.36	0.24	0.03
β_6_ (Castrove)	0.13	0.02	0.20	0.09	0.06	-	0.16	0.07	0.09
β_7_ (Sanxenxo)	0.18	0.28	−0.15	0.03	0.36	0.21	-	0.09	0.07
β_8_ (Corón)	0.12	0	0	0.06	0.19	0.07	0.06	-	0.15
**β_9_ (Torrequint**á**ns)**	**0.17**	**0.05**	**0.07**	**0.42**	**0.04**	**0.13**	**0.08**	**0.23**	-

**Table 7.** RMSE and MAE obtained with the MLR models.

**Station**	**RMSE (%)**	**MAE (%)**
Castrelo	26.80	28.40
Simes	25.55	22.73
A Armenteira	28.40	31.91
Pé Redondo	28.38	41.73
A Lanzada	27.47	35.70
Castrove	33.81	48.30
Sanxenxo	26.98	32.93
Corón	29.78	45.63

**Torrequint**á**ns**	**26.52**	**30.36**

**Table 8.** RMSE and MAE obtained with MICE.

	**Castrelo**		**Simes**		**A Armenteira**		**Pé Redondo**		**A Lanzada**		**Castrove**		**Sanxenxo**		**Corón**		**Torrequintáns**	

**Iteration**	**RMSE (%)**	**MAE (%)**	**RMSE (%)**	**MAE (%)**	**RMSE (%)**	**RMSE (%)**	**RMSE (%)**	**MAE (%)**	**RMSE (%)**	**MAE (%)**	**RMSE (%)**	**MAE (%)**	**RMSE (%)**	**MAE (%)**	**RMSE (%)**	**MAE (%)**	**RMSE (%)**	**MAE (%)**
1	12.74	11.03	11.93	10.26	13.29	12.57	13.39	16.71	12.57	11.98	15.86	21.11	12.41	14.22	13.79	13.94	12.86	13.36
2	12.60	10.70	11.79	10.46	13.33	12.46	13.26	16.56	12.46	12.08	15.58	20.57	12.43	14.19	14.00	13.85	12.32	13.25
3	12.60	10.72	12.27	10.59	13.46	12.67	13.41	17.03	12.67	12.12	15.72	20.62	12.43	14.16	14.08	14.71	12.44	13.03
4	12.68	10.72	12.00	11.12	13.64	12.59	13.12	16.20	12.59	12.21	15.69	20.41	12.64	14.75	14.03	13.92	12.63	13.40
5	12.55	10.67	11.80	10.33	13.15	12.35	13.56	16.97	12.35	11.69	15.66	20.26	12.45	14.49	13.87	13.61	12.53	13.35

Average	12.63	10.77	11.96	10.55	13.37	12.53	13.35	16.69	12.53	12.02	15.70	20.59	12.47	14.36	13.95	14.01	12.56	13.28

**Table 9.** Results of the three models in terms of RMSE.

**Station**	**MICE (%)**	**MLR (%)**	**IDW (%)**
Castrelo	12.74	26.80	29.13
Simes	12.27	25.55	31.55
A Armenteira	13.64	28.40	37.33
Pé Redondo	13.56	28.38	29.67
A Lanzada	12.67	27.47	30.41
Castrove	15.86	33.81	34.34
Sanxenxo	12.64	26.98	33.25
Corón	14.08	29.78	32.69

**Torrequint**á**ns**	**12.86**	**26.52**	**26.73**

**Table 10.** Results of the three models in terms of MAE.

**Station**	**MICE (%)**	**MLR (%)**	**IDW (%)**
Castrelo	11.03	28.40	22.89
Simes	10.59	22.73	35.81
A Armenteira	12.53	31.91	46.51
Pé Redondo	16.97	41.73	37.53
A Lanzada	12.2	35.70	24.31
Castrove	21.11	48.30	50.29
Sanxenxo	14.75	32.93	32.54
Corón	14.71	45.63	29.12

**Torrequint**á**ns**	**13.36**	**30.36**	**28.69**
